# 290 Admission Inflammatory Cytokine Levels Are Predictive of Fluid Resuscitation Requirements in Thermally Injured Patients

**DOI:** 10.1093/jbcr/irad045.265

**Published:** 2023-08-29

**Authors:** Edward J Kelly, Bonnie C Carney, John Keyloun, Lauren T Moffatt, Jeffrey W Shupp, Shawn Tejiram

**Affiliations:** Firefighter’s Burn and Surgical Research Laboratory, Washington, District of Columbia; Firefighters’ Burn and Surgical Research Laboratory, Medstar Health Research Institute, Georgetown University Medical Center, Washington, District of Columbia; Medstar Health, Washington DC, District of Columbia; Medstar Health, Washington DC, District of Columbia; Firefighters’ Burn and Surgical Research Laboratory, MedStar Health Research Institute, Georgetown Univeristy School of Medicine, Washington, District of Columbia; MedStar Washington Hospital Center, Washington, District of Columbia

## Abstract

**Introduction:**

Resuscitation of patients with major thermal injury is accomplished with crystalloid infusion-based injury size and patient weight ultimately titrated based on patient response. Estimations of fluid requirements can be imprecise and predicting responsiveness lacks precision. Previous work has demonstrated perturbations in both proinflammatory and anti-inflammatory cytokines in burn patients. However, there is a paucity of literature examining the impact of these perturbations and their usefullness to help predict resuscitation needs. This study sought to examine patient specific cytokine levels which may provide better insight into resuscitation requirements.

**Methods:**

Burn injured patients presenting to a regional center over six years were prospectively enrolled in this observational clinical trial. Blood samples were collected on admission. Plasma cytokine levels (IL-1b, IL-6, IL-10, IL-12p70 and TNF-a) were quantified by ELISA. Crystalloid resuscitation volumes during the first 48 hours were assessed for correlation with cytokine levels after adjusting for weight and %TBSA.

**Results:**

Thirty-eight patients were included in the analysis. Mean total body surface area (TBSA) burned was 26.7±14.4%. Elevated levels of IL-6 significantly correlated with normalized resuscitation volumes (ml/kg/%TBSA) from 8-24hrs and 24-48hrs (r=0.52, p< 0.001; r=0.41, p< 0.01). Elevated levels of IL-10 were also significantly correlated with normalized crystalloid volumes from 8-24hrs and 24-48hrs (r=0.55, p< .01; r=0.46, p< 0.001). Elevated levels of TNF-a also correlated with normalized crystalloid volumes from 8-24hrs and 24-48hrs (r=0.60, p< 0.0001; r=0.57, p< 0.001).

**Conclusions:**

Predicting resuscitation volumes (and preventing over excessive crystalloid administration—fluid creep) is an integral aspect of burn injury management and there is a need for more objective methods for measurement. Phenotyping patients based on admission cytokine levels employing point of care assays may be a novel aid in predicting resuscitation needs and avoiding fluid creep.

**Applicability of Research to Practice:**

Proper burn resuscitation is an integral aspect in the care of the burn-injured patient. Establishing adjuncts to help estimate fluid requirements or risk of over-resuscitation could be integral in establishing more individualized care.

